# Toxicities and Quality of Life during Cancer Treatment in Advanced Solid Tumors

**DOI:** 10.3390/curroncol30100665

**Published:** 2023-10-19

**Authors:** Eun Mi Lee, Paula Jiménez-Fonseca, Rocio Galán-Moral, Sara Coca-Membribes, Ana Fernández-Montes, Elena Sorribes, Esmeralda García-Torralba, Laura Puntí-Brun, Mireia Gil-Raga, Juana Cano-Cano, Caterina Calderon

**Affiliations:** 1 Faculty of Psychology, University of Barcelona, 08007 Barcelona, Spain; 2 Department of Medical Oncology, Hospital Universitario Central de Asturias, ISPA, 33011 Oviedo, Spain; 3 Department of Medical Oncology, Hospital General Universitario de Ciudad Real, 13005 Madrid, Spain; 4 Department of Medical Oncology, Hospital Universitario de Canarias, 38320 Tenerife, Spain; 5 Department of Medical Oncology, Complejo Hospitalario Universitario de Ourense, 32005 Ourense, Spain; 6 Department of Medical Oncology, Hospital Universitario Morales Meseguer, 30008 Murcia, Spain; 7 Department of Medical Oncology, Consorcio Sanitario del Maresme, 08304 Mataró, Spain; 8 Department of Medical Oncology, Hospital General Universitario de Valencia, 46014 Valencia, Spain

**Keywords:** toxicities, symptoms, advanced cancer, adverse effects, quality of life

## Abstract

The purpose of the study was to identify subgroups of advanced cancer patients who experienced grade 3–4 toxicities as reported by their oncologists as well as identify the demographic, clinical, and treatment symptom characteristics as well as QoL outcomes associated with distinct profiles of each patient. A prospective, multicenter, observational study was conducted with advanced cancer patients of 15 different hospitals across Spain. After three months of systemic cancer treatment, participants completed questionnaires that evaluated psychological distress (BSI-18), quality of life (EORTC QLQ-C30) and fatigue (FAS). The most common tumor sites for the 557 cancer patients with a mean age of 65 years were bronchopulmonary, digestive, and pancreas. Overall, 19% of patients experienced high-grade toxicities (grade 3–4) during treatment. Patients with recurrent advanced cancer, with non-adenocarcinoma cancer, undergoing chemotherapy, and a showing deteriorated baseline status (ECOG > 1) were more likely to experience higher toxicity. Patients who experienced grade 3–4 toxicities during cancer treatment had their treatment suspended in 59% of the cases. Additionally, 87% of the patients had a dose adjustment or a cycle delayed in their treatment due to a high risk of dying during treatment. Future research should focus on identifying interventions to reduce high-grade toxicities and improve quality of life in cancer patients.

## 1. Introduction

The treatment of patients with advanced cancer is focused on palliative cytotoxic chemotherapy, targeted therapy, and immunotherapy with the use of checkpoint inhibitors [1,2]. Targeted therapy and immunotherapy differ from conventional chemotherapy in terms of mechanism of action, route of administration (often oral), time of response, toxicity profile, and cost [2,3]. Although the goals of all these treatments are to improve quality of life, reduce symptom burden, and prolong survival, some patients may experience toxic effects that significantly impact their quality of life [3,4]. In order to maximize the benefit and minimize the risks associated with these treatments, it is essential to understand the characteristics and consequences of oncologic therapies in the growing population of patients with advanced cancer [5,6]. When planning and continuing cytotoxic treatment, it is crucial to integrate toxicity prevention as well as maintain or improve patients ‘quality of life [1]. Unfortunately, toxicities experienced by patients are frequent difficult to predict and prevent.

It is indispensable for oncologists to improve the prevention and management of the side effects of cancer treatment and concomitantly gain the ability to predict severe (grade 3–4) toxicity [5,7,8]. Anticipated prediction of toxicity would allow more effective counseling, prophylaxis, and guidance in dose administrations and treatment intervals [7,9,10]. Numerous studies have suggested that specific clinical factors, such as previous neoplasia, non-adenocarcinoma cancer, record of receiving chemotherapy, and poorer baseline status, are associated with a higher risk of experiencing severe toxicity during cancer treatment [11,12,13]. For example, a study by Nguyen et al. (2022) examined risk factors for chemotherapy-induced toxicity in breast cancer patients and revealed that individuals with a history of previous neoplasm and record of receiving chemotherapy had a significantly higher likelihood of developing grade 3–4 toxicity [11]. In addition, another study by Dumenil et al. (2018) evaluated the incidence and risk factors of severe toxicity in non-small cell lung cancer patients receiving chemotherapy, and the results indicated that patients with histology other than adenocarcinoma and poor functional status substantially showed a higher risk of grade 3–4 toxicity [12]. Correspondingly, gastric cancer patients receiving chemotherapy with poor functional status had a higher risk of grade 3–4 toxicity [13]. These results indicate that clinical factors such as tumor, histology, treatment, or presenting poor functional status may be risk factors for developing grade 3–4 toxicity. It is vital in oncological treatment that patients with advanced cancer try to prolong life even though it may not be curative, since toxicities can be severe and long lasting, causing a negative impact on patients’ quality of life.

The assessment of quality of life is an essential aspect in patients with advanced cancer; however, traditional assessment criteria in cancer clinical research often overlook this crucial aspect and focus more on tumor control rate, overall survival, or disease-free survival [14,15]. Quality of life refers to a global health status as well as changes in emotional, physical, social, and cognitive functioning [16,17]. Quality of life can be affected by various patient-specific, disease-specific, and oncology treatment-specific factors [17]. Despite its importance, the assessment of quality of life remains as a challenge in many cases as cancer symptoms might worsen before any improvement, given that both the mental and physical states of patients may vary in the pre-treatment stage. Moreover, due to the heterogeneity of cancer diseases and their clinical manifestations, assessing quality of life after treatment in different cancer patients remains difficult to generalize. Another common side effect reported by cancer patients during and after treatment is fatigue, which can be caused by the tumor itself or by the oncology treatment. Numerous studies have found that the presence of severe (grade 3–4) toxicity lays a significant impact on quality of life and fatigue perception of patients with advanced cancer [18,19,20]. These circumstances can affect patients’ ability to perform their daily activities as well as their ability to maintain healthy social and emotional relationships, which can lead to a deterioration in their emotional state.

To achieve an effective treatment in advanced cancer, it is key to understand the characteristics and consequences of oncology therapies as well as to evaluate their impact on patients’ quality of life. In this way, the benefits can be maximized, and the associated risks can be minimized. Therefore, the objectives of the study were to identify patients with advanced cancer who have experienced grade 3–4 toxicities as reported by their oncologists, analyze the relationship between patients’ grade 3–4 toxicities with demographic and clinical characteristics, quality of life (functional status and symptoms), fatigue, and emotional state.

## 2. Materials and Methods

### 2.1. Study Design and Population

**Figure 1:**
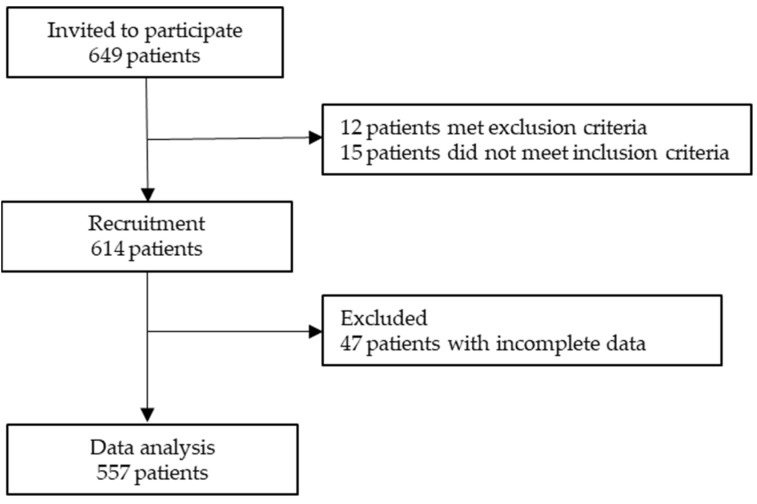
Flow chart comprising inclusion and exclusion criteria of participants in the study.

A prospective cross-sectional study was conducted across 15 medical oncology departments at various hospitals throughout Spain between February 2020 and March 2023. The study aimed to gather data on patients with advanced cancer who were not suitable for curative therapy. Patients were recruited during their initial visit to the medical oncologist, where they were informed about their diagnosis, disease stage, and available systemic antineoplastic treatments. In order to be eligible for the study, participants had to be 18 years of age or older, with a histologically confirmed advanced cancer, and not be a candidate for surgery or other curative therapies. Patients were excluded if their physical condition, age, or comorbidity contraindicated antineoplastic treatment according to the attending oncologist’s opinion. Additionally, patients who had received treatment for another type of advanced cancer within the past two years or those with underlying medical, sociological, family, or personal conditions that could hinder their participation in the study were excluded. Patients with cognitive impairment, severe deterioration of their general status, or those unable to understand or reason through the questionnaires were also excluded. The study was approved by the Ethics Review Committee of each participating Institution and by the Spanish Agency of Medicines and Health Products (AEMPS; identification code: ES14042015). All participants provided informed consent before enrollment in the research study. The data were collected through questionnaires and interviews as well as from the medical records. The data collection process was standardized across all participating hospitals, and patients’ data were obtained from the institutions where they received treatment. Participation was voluntary and anonymous, which did not affect patients’ care. Medical oncologists updated and collected data using a web-based platform: www.neoetic.es (accessed on 20 July 2023). A flow chart comprising the inclusion and exclusion criteria for participants is given in Figure 1.

### 2.2. Description of Variables

Participants’ sociodemographic characteristics were collected using a standardized self-report form. The information related to participants’ disease was obtained by reviewing their medical history.

To evaluate toxicity and treatment response, oncologists answered a series of questions three months after starting cancer treatment. The oncologists indicated whether the patient had experienced any grade 3–4 toxicities that had an impact on their quality of life, and based on this information, patients were classified as having or not having grade 3–4 toxicities. Oncologists assessed tumor response to treatment using RECIST 1.1 criteria (Response Evaluation Criteria in Solid Tumors) [21]. These criteria are used to measure changes in tumor size in response to treatment as “complete”, “partial”, “stable”, or “progression”. This classification is important, since it gives an evaluation of the treatment efficacy and can guide decisions regarding treatment continuation or modification. During this process, oncologists also answered a series of questions related to treatment evaluation of the patients (Table A1).

The patients completed a series of questionnaires (EORTC QLQ-C30, FAS, and BSI-18) three months after starting their systemic cancer treatment.

The European Organization for Research and Treatment of Cancer Quality of Life Questionnaire (EORTC QLQ-C30) contains 30 items comprising three subscales: functioning, symptoms, and health status [16]. The response choices range from 1 (not at all) to 4 (very much), except for the health status scale, where responses range from 1 (very poor) to 7 (excellent). All scale scores are linearly transformed into a 0–100 scale. Higher scores on the functioning scales and health status scale represent a higher level of functioning or QoL. For the symptom scales, the higher the score, the greater the symptom burden. The Spanish version of the questionnaire has demonstrated good reliability with a Cronbach’s alpha of 0.85 [22].

The Fatigue Assessment Scale (FAS) [23] is a self-report questionnaire consisting of 10 items that measures the physical and psychological aspects of fatigue. Each item is rated on a 5-point Likert scale ranging from “1 = never” to “5 = always”. A higher score defines more symptoms of mental and/or physical fatigue. The Cronbach’s alpha coefficient was 0.80 [23].

The Brief Symptom Inventory (BSI-18) is a self-report questionnaire compromised of 18 items that evaluate a respondent’s mental well-being [24]. The scale includes symptoms of depression (dysphoric mood, anhedonia, and self-deprecation), anxiety (nervousness, tension, and apprehension), and somatization (distress caused by the perception of bodily dysfunction). Each item is rated on a 5-point Likert scale with scores ranging from 0 (representing “not at all”) to 4 (representing “extremely”). The raw score is converted to T-scores based on sex-specific normative data. The Spanish version of the questionnaire has demonstrated good reliability with a Cronbach’s alpha of 0.88 [25].

### 2.3. Statistical Methods

The mean, standard deviation (SD), number (N), and percentage (%) were used to report descriptive statistics for demographic and other variables. To compare patients with and without toxicities of grade 3–4 in terms of sociodemographic and clinical characteristics, a chi-square test and one-way ANOVA were performed. Eta-squared was used to determine the effect size of differences with a range between 0 and 1. A value of η^2^ ~ 0.1 indicates a small effect size, η^2^ ~ 0.06 indicates a medium effect size, and η^2^ > 0.14 indicates a large effect size [26]. Statistical analysis was conducted using the Statistical Package for Social Sciences (SPSS) version 26.0 for Windows (SPSS Inc., Chicago, IL, USA). All tests were two-sided, and the significance level was set at *p* < 0.05.

## 3. Results

### 3.1. Sociodemographic and Clinical Characteristics

Out of the 557 patients who met the eligibility criteria, the majority of those included in the study were male (54%), aged ≥65 years (55%), and were either married or in a committed relationship (68%). The most common tumor sites were bronchopulmonary (28%), digestive (34%), and pancreas (11%), with a high proportion of patients presenting metastatic disease at the time of diagnosis (70% versus 30%). The primary treatment method employed was chemotherapy (56%), which was followed by targeted therapies (6%) and immunotherapy (6%). The majority of participants had an ECOG (Eastern Cooperative Oncology Group) status of >1 at the time of diagnosis (64%) with more than half of the cases (73%) having an estimated survival rate of 18 months.

### 3.2. Toxicity Profiles and Clinical Demographic Characteristics

During treatment, 19% (*n* = 109) of patients experienced grade 3–4 toxicities as reported by their oncologists. Upon analyzing the relationship between toxicity and the clinical and demographic characteristics of the study population, patients with a non-adenocarcinoma (as small cell, squamous cell, neuroendocrine, sarcoma, germ cells or melanoma) (*X*^2^ = 7.2508, *p* = 0.007), with a recurrent advanced cancer (*X*^2^ = 4.763, *p* = 0.029), undergoing chemotherapy treatment (*X*^2^ = 11.398, *p* = 0.010), and with a deteriorated baseline status (ECOG > 1) (*X*^2^ = 7.365, *p* = 0.007) showed higher levels of toxicity. However, there was no statistically significant relationship observed between toxicity and demographic characteristics (sex, age, marital status, educational level, employment, cancer stage, or estimated survival) (Table 1).

### 3.3. Medical Follow-Up

During medical follow-up, while undergoing treatment, patients who experienced grade 3–4 toxicities had their treatment suspended (59% of cases), while 87% had their dose adjusted or cycle delayed. The radiological response to chemotherapy had been evaluated at 3 months in 99% of the patients, and the tumor response rates were as follows: 8% had complete responses, 43% had partial responses, 27% had stable disease, and 23% experienced tumor progression. Furthermore, 50% of the patients had their treatment discontinued. In retrospect, oncologists would have preferred a less toxic (19%) and/or aggressive treatment (4%). Additionally, 20% of oncologists believed that the treatment might have improved patients’ survival but potentially at the cost of a reduced quality of life and increased toxicity. Patients who experienced grade 3–4 toxicities during treatment had a higher risk of dying during the first 3 months of treatment (33%, *n* = 35). Meanwhile, patients who did not experience grade 3–4 toxicities during the first 3 months on treatment continued with their prescribed treatment (96% of patients), 73% did not have to delay doses or cycles of treatment, 7% of patients underwent cycles of chemotherapy in which medication doses were adjusted, and 33% discontinued first-line treatment. In addition, 55% of the patients achieved a partial response to treatment, and 24% had a stable disease (Table 2).

**Figure 2:**
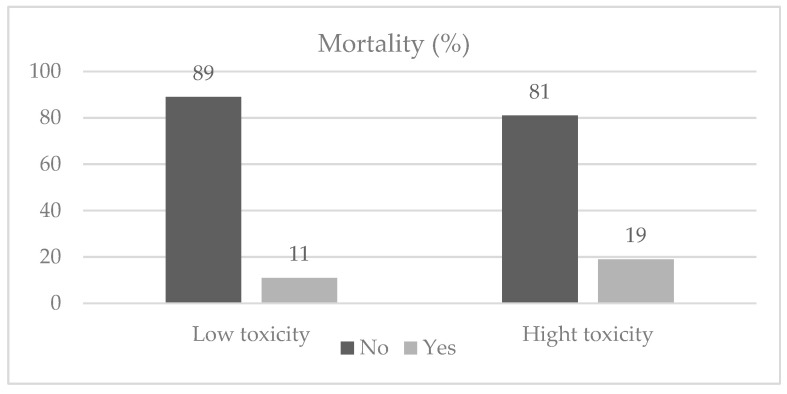
Mortality among study participants.

In retrospect, 89% of oncologists would like to apply the same treatment, and 70% of the oncologists believed that oncological treatment reduced symptoms, improved quality of life, and increased survival. Unfortunately, 12.6% of the patients (*n* = 70) passed away (see Figure 2).

### 3.4. Toxicity Profiles and Psychosocial Characteristics

In search of relationships between toxicity profiles and psychosocial symptoms, quality of life, and fatigue, results showed that cancer patients with higher toxicity levels (grade 3–4) demonstrated lower scores on the functional scale (*M* = 65.6 vs. *M* = 57.9; *η*^2^ = 0.025), higher scores on the symptom scale (*M* = 23.5 vs. *M* = 33.1; *η*^2^ = 0.032), higher levels of physical fatigue (*M* = 13.2 vs. *M* = 16.1; *η*^2^ = 0.052), and higher levels of mental fatigue (*M* = 11.5 vs. *M* = 16.1; *η*^2^ = 0.016). However, there were no statistically significant differences in psychological distress, as shown in Table 3.

## 4. Discussion

Based on the literature review, the current study has the largest number of Spanish patients with recent advanced cancer diagnoses to date with a specific design to investigate the incidence of high-grade (3–4) toxicity levels in advanced cancer patients and its possible associations with demographic, clinical, and psychological factors.

In the study population, 19% of the patients (n = 109) experienced high-grade toxicities (grade 3–4) during their cancer treatment as reported by their oncologists. Patients with certain clinical characteristics, such as recurrent advanced cancer, non-adenocarcinoma cancer, undergoing chemotherapy, and a deteriorated baseline status (ECOG >1), were more likely to experience higher levels of toxicity. However, no statistically significant relationships were found between toxicity and other factors, such as demographic (sex, age, marital status, educational level, employment, cancer stage, or estimated survival) and psychological factors. This finding is consistent with previous research that has identified various clinical and demographic factors that may influence the incidence and severity of cancer treatment-related toxicities [3,27,28]. For instance, prior studies have shown that patients with a history of previous neoplasm, non-adenocarcinoma histology, and those with poor performance status had a significantly higher risk of grade 3–4 toxicity as well as higher rates of hospital admissions [8,11,28,29]. Patients with cancers of histologies different from adenocarcinoma, especially those of non-epithelial lineage such as germ cell tumors, sarcomas, and small and large cell neuroendocrine carcinomas, often undergo more intensive therapeutic regimens and are, therefore, associated with a higher risk of toxicity. In contrast, those with adenocarcinoma may possess certain characteristics or molecular profiles rendering them less susceptible to high-grade toxicities compared to patients with other cancer types. Additionally, bronchopulmonary cancers are more likely to cause treatment-related toxicities than other types of cancer [30,31]. Overall, the findings of this study highlight the importance of considering patient-specific factors when assessing and managing treatment-related toxicities in cancer patients [27].

Moreover, patients who have experienced grade 3–4 toxicities were more likely to suspend or delay their cycles or doses of cancer treatment. In hindsight, 19% of oncologists would have preferred a less toxic and/or aggressive treatment (4%). Furthermore, 20% of oncologists believed that the treatment improved patients’ survival at the expense of a worse quality of life and high toxicity. These findings suggest that the severity of toxicities during cancer treatment can impact patients’ quality of life and survival outcomes. Also, patients who have experienced grade 3–4 toxicities during treatment had a higher risk of dying during treatment (33%, n = 35). These findings are consistent with previous research indicating that treatment-related toxicities can impact outcomes in cancer patients [7,32]. Several studies have demonstrated that patients experiencing severe adverse events during cancer treatment had a higher risk of treatment interruption, discontinuation, and death [32,33,34]. In addition, these toxicities can affect patients’ quality of life and increase healthcare costs [35,36,37]. Therefore, managing toxicity is an important aspect of cancer treatment to improve patient outcomes. Various interventions such as supportive care measures and dose adjustments can reduce the risk and severity of toxicities during cancer treatment. Nonetheless, our findings revealed no significant differences in psychological distress between metastatic cancer patients with high and low toxicity profiles. This suggests that individuals from both groups might encounter comparable levels of emotional and psychological distress. Such an observation underscores the idea that psychological distress remains a significant concern for metastatic cancer patients irrespective of the extent of treatment-related side effects.

Finally, it has been observed that patients who experienced higher levels of toxicity had deteriorated quality of life, increased fatigue, and more physical and functional symptoms. These findings were consistent with previous studies that identified a relationship between toxicities and quality of life in advanced cancer patients [38,39,40]. For example, in a study by Dueck et al. (2015), cancer patients who experienced severe toxicities had worse quality of life and physical functioning overall [40]. Moreover, a study by Fabi et al. (2020) found that cancer patients with higher levels of fatigue were at increased risk of experiencing treatment-related toxicities [39]. These findings underscore the importance of managing treatment toxicities to improve the quality of life and overall well-being of patients with advanced cancer.

This study offers significant clinical insights by highlighting risk factors associated with severe toxicities in advanced cancer patients, such as a recurrent advanced cancer and specific cancer types. These findings equip healthcare professionals with vital knowledge to better anticipate and manage potential serious complications and side effects, thereby enhancing clinical care and decision-making processes. These findings lay the groundwork for subsequent research and intervention strategies, aiming to decrease the occurrence of severe toxicities and enhancing the quality of life for oncology patients. Notably, the strengths of this study lie in its prospective, multicenter design, which encompasses a broad patient sample from various Spanish hospitals. The application of standardized questionnaires for assessing psychological distress, quality of life, and fatigue lends further credibility to the findings. In essence, this study significantly advances the understanding and management of cancer treatment toxicities, ultimately serving the best interests of patients.

The study presents both strengths and limitations. A major strength lies in its distinction as the first study in Spain to identify advanced cancer patients experiencing grade 3–4 toxicities and to examine the association with demographic and clinical characteristics, quality of life (both functional status and symptoms), fatigue, and emotional state. However, there were several limitations in the current study. First, due to the cross-sectional design, it was not possible to determine the directionality of the relationships observed. Therefore, future longitudinal cohort studies would be needed to confirm the findings from this study. Second, the study population was diverse and integrated by patients with various tumor types and treatment regimens, which may have limited the generalizability of the results. Finally, while our sample size is representative of the Spanish population, the findings might not be directly applicable to patients with resectable cancer. Moreover, care should be taken when extrapolating these results to different cultural or societal contexts.

## 5. Conclusions

In conclusion, the current study highlights the impact of cancer treatment-related toxicities in patient outcomes, emphasizing the need for personalized toxicity management. Patients with clinical characteristics, such as previous neoplasm, non-adenocarcinoma cancer, undergoing chemotherapy treatment, and deteriorated baseline status, are more likely to experience higher levels of toxicity.

Additionally, patients who have experienced grade 3–4 toxicities had a higher likelihood of treatment suspension or delay, which can impact patients’ survival. The study also found that patients who experienced higher levels of toxicity had worse quality of life, increased fatigue, and more physical and functional symptoms, further emphasizing the need for toxicity management to improve patients’ well-being. Healthcare providers must consider patient-specific factors when assessing and managing treatment-related toxicities to minimize treatment interruption, improve quality of life, and enhance patients’ outcomes.

## Figures and Tables

**Table 1:** Differences in demographic and clinical characteristics among the toxicities’ profiles (n = 557).

Variables	Total Sample n (%) 557 (100%)	Low Toxicity n (%) 451 (81%)	High Toxicity n (%) 106 (19%)	*X* ^2^	*p* Value
Sex				3.218	0.073
Male	298 (54)	233 (52)	65 (61)		
Female	259 (46)	218 (48)	41 (39)		
Age				0.322	0.570
<65 y	246 (45)	199 (44)	50 (47)		
≥65 y	308 (55)	252 (56)	56 (53		
Marital status				0.335	0.563
Partnered	381 (68)	306 (68)	75 (71)		
No partnered	176 (32)	145 (32)	31 (29)		
Educational level				0.086	0.769
Primary	233 (42)	190 (42)	43 (41)		
≥High school	324 (58)	261 (58)	63 (59)		
Employment				0.024	0.878
With employment	259 (47)	209 (46)	50 (47)		
Without employment	298 (54)	242 (54)	56 (53)		
Tumor site				5.923	0.205
Bronchopulmonary	158 (28)	135 (30)	23 (22)		
Digestive	131 (24)	101 (22)	30 (28)		
Pancreas	60 (11)	49 (11)	11 (10)		
Breast	48 (9)	42 (9)	6 (6)		
Others	160 (29)	124 (28)	36 (34)		
Histology				**7.250**	**0.007**
Adenocarcinoma	391 (70)	328 (73)	63 (59)		
Others	166 (30)	123 (27)	43 (41)		
Stage				0.780	0.377
Locally Advanced	119 (21)	93 (21)	26 (25)		
Metastatic Dis. (IV)	438 (79)	358 (79)	80 (75)		
Recurrent advanced cancer				**4.763**	**0.029**
Yes	92 (17)	82 (18)	10 (9)		
No	465 (84)	369 (82)	96 (90)		
Type of treatment				**11.398**	**0.010**
Chemo monotherapy	314 (56)	240 (53)	74 (70)		
Chemo combined	178 (32)	151 (34)	27 (26)		
Immunotherapy	31 (6)	29 (6)	2 (2)		
Targeted therapies	34 (6)	31 (7)	3 (3)		
ECOG				**7.365**	**0.007**
0	200 (36)	174 (39)	26 (25)		
1 or more	357 (64)	277 (61)	80 (75)		
Estimated Survival				0.073	0.787
<18 months	404 (73)	326 (72)	78 (74)		
≥18 months	153 (27)	125 (28)	28 (26)		

Bold values indicate the significant at 5% level.

**Table 2:** Differences in medical follow-up (*n* = 557).

Variables	Total 557 (100%)	Low Toxicity 451 (81%)	High Toxicity 106 (19%)	*X* ^2^	*p* Value
Treatment suspension				**116.003**	**0.001**
No	493 (89)	431 (96)	62 (59)		
Yes	64 (12)	20 (4)	44 (41)		
Delay doses or cycles				**128.291**	**0.001**
No	342 (61)	328 (73)	14 (13)		
Yes	215 (39)	123 (27)	92 (87)		
Doses adjusted or cycle delayed of chemotherapy				**6.083**	**0.014**
No	34 (6)	33 (7)	1 (1)		
Yes	523 (94)	418 (93)	105 (99)		
Suspension of first line treatment				**10.384**	**0.001**
No	354 (64)	301 (67)	53 (50)		
Yes	203 (36)	150 (33)	53 (50)		
Has progressed in first line of treatment				**4.509**	**0.034**
No	389 (70)	324 (72)	65 (61)		
Yes	168 (30)	127 (28)	41 (39)		
Best response by RECIST 1.1				5.180	0.159
Complete response	30 (6)	22 (5)	8 (8)		
Partial response	277 (52)	232 (55)	45 (43)		
Stable disease	129 (24)	101 (24)	28 (27)		
Progression	95 (18)	71 (17)	24 (23)		
In retrospect, continuance of same treatment				**31.499**	**0.001**
Same	476 (86)	700 (89)	76 (72)		
Less toxic	38 (4)	18 (4)	20 (19)		
Less aggressive	20 (4)	16 (4)	4 (4)		
Palliative	23 (4)	17 (4)	6 (6)		
In retrospect, treatment had,				**55.028**	**0.001**
No benefit	81 (15)	66 (15)	15 (14)		
Benefit in symptoms and QoL	55 (10)	46 (10)	9 (9)		
Benefit in survival	40 (7)	19 (4)	21 (20)		
Benefit in symptoms QoL, and survival	365 (66)	314 (70)	51 (48)		
deterioration	16 (3)	6 (1)	10 (9)		
Death				**4.730**	**0.030**
No	487 (87)	401 (89)	86 (81)		
Yes	70 (13)	50 (11)	20 (19)		

Bold values indicate the significant at 5% level.

**Table 3:** Differences in psychosocial distress, quality of life, fatigue, and toxicity (n = 557).

	Low Toxicity 283 (56%)		High Toxicity 225 (44%)				
Variables	Mean	SD	Mean	SD	F	*p*	Eta-Squared
Psychological distress (BSI)							
Depression	61.5	6.6	62.2	6.1	0.576	0.448	---
Anxiety	62.3	7.4	62.6	8.1	0.088	0.767	---
Somatization	64.7	7.4	66.5	7.3	2.972	0.086	---
Quality of life (EORTC)							
Functional scale	65.6	16.7	57.9	20.9	9.667	0.002	0.025
Symptom scale	23.5	18.3	33.1	22.1	12.754	0.001	0.032
Health status	59.9	265.6	59.3	28.9	0.031	0.861	---
Fatigue (FAS)							
Physic fatigue	13.2	4.2	16.1	5.3	20.880	0.001	0.052
Mental fatigue	11.5	3.6	12.9	4.4	6.329	0.012	0.016

*Abbreviation:* BSI: Brief Symptom Inventory; EORTC-QoL-QLQ-C30 = European Organization for Research and Treatment of Cancer Quality of Life Questionnaire; FAS: Fatigue Assessment Scale; SD: standard deviation.

## Data Availability

The datasets generated during and analyzed during the current study are not publicly available for reasons of privacy. They are however available (fully anonymized) from the corresponding author on reasonable request.

## Appendix A

**Table A1:** List of 15 centers participants in the study.

(1)	Hospital Universitario de Canarias, Tenerife
(2)	Hospital Universitario La Paz, Madrid
(3)	Hospital Universitario Central de Asturias, Oviedo
(4)	Complejo Hospitalario Universitario de Ourense, Orense
(5)	Hospital Universitario Infanta Leonor, Madrid
(6)	Consorcio Hospital General Universitario de Valencia, Valencia
(7)	Hospital General Virgen de la Luz, Cuenca
(8)	Hospital Provincial de Castellón, Castellón
(9)	Hospital General Universitario de Elche, Elche
(10)	Hospital Universitario Clínico San Carlos, Madrid
(11)	Hospital San Pedro de Alcántara, Cáceres
(12)	Hospital Universitario Morales Meseguer, Murcia
(13)	Hospital Quironsalud Sagrado Corazón de Sevilla, Sevilla
(14)	Hospital General Universitario Santa Lucia, Cartagena
(15)	Hospital General Universitario de Ciudad Real, Ciudad Real
